# Diversities and potential biogeochemical impacts of mangrove soil viruses

**DOI:** 10.1186/s40168-019-0675-9

**Published:** 2019-04-11

**Authors:** Min Jin, Xun Guo, Rui Zhang, Wu Qu, Boliang Gao, Runying Zeng

**Affiliations:** 1 State Key Laboratory Breeding Base of Marine Genetic Resource, Third Institute of Oceanography, Ministry of Natural Resources, Xiamen, China; 2 State Key Laboratory of Marine Environmental Science, College of Ocean and Earth Sciences, Institute of Marine Microbes and Ecospheres, Xiamen University, Xiamen, China; 3 Fujian Collaborative Innovation Center for Exploitation and Utilization of Marine Biological Resources, Xiamen, China

**Keywords:** Mangrove soil, Viruses, Viromes, Carbon cycling, Auxiliary metabolic genes

## Abstract

**Background:**

Mangroves are ecologically and economically important forests of the tropics. As one of the most carbon-rich biomes, mangroves account for 11% of the total input of terrestrial carbon into oceans. Although viruses are considered to significantly influence local and global biogeochemical cycles, little information is available regarding the community structure, genetic diversity and ecological roles of viruses in mangrove ecosystems.

**Methods:**

Here, we utilised viral metagenomics sequencing and virome-specific bioinformatics tools to study viral communities in six mangrove soil samples collected from different mangrove habitats in Southern China.

**Results:**

Mangrove soil viruses were found to be largely uncharacterised. Phylogenetic analyses of the major viral groups demonstrated extensive diversity and previously unknown viral clades and suggested that global mangrove viral communities possibly comprise evolutionarily close genotypes. Comparative analysis of viral genotypes revealed that mangrove soil viromes are mainly affected by marine waters, with less influence coming from freshwaters. Notably, we identified abundant auxiliary carbohydrate-active enzyme (CAZyme) genes from mangrove viruses, most of which participate in biolysis of complex polysaccharides, which are abundant in mangrove soils and organism debris. Host prediction results showed that viral CAZyme genes are diverse and probably widespread in mangrove soil phages infecting diverse bacteria of different phyla.

**Conclusions:**

Our results showed that mangrove viruses are diverse and probably directly manipulate carbon cycling by participating in biomass recycling of complex polysaccharides, providing the knowledge essential in revealing the ecological roles of viruses in mangrove ecosystems.

**Electronic supplementary material:**

The online version of this article (10.1186/s40168-019-0675-9) contains supplementary material, which is available to authorized users.

## Background

Viruses are the most abundant biological entities on earth; they are virtually present in all ecosystems [1, 2]. By lysing their hosts, viruses control host abundance and affect the structure of host communities [3]. Viruses also influence their host diversity and evolution through horizontal gene transfer, selection for resistance and manipulation of bacterial metabolisms [4–7]. Importantly, viruses affect local and global biogeochemical cycles through the release of substantial amounts of organic carbon and nutrients from hosts and assist microbes in driving biogeochemical cycles with auxiliary metabolic genes (AMGs) [8–11].

The presence of AMGs in viruses has been described previously; AMGs are presumed to augment viral-infected host metabolism and facilitate production of new viruses [4, 12]. AMGs are most extensively explored in marine cyanophages and include genes involved in photosynthesis, carbon turnover, phosphate uptake and stress response [13–16]. Cultivation-independent metagenomic analysis of viral communities has identified additional AMGs that are involved in motility, central carbon metabolism, photosystem I, energy metabolism, iron–sulphur clusters, anti-oxidation and sulphur and nitrogen cycling [10, 17–22]. Interestingly, a recent analysis of Pacific Ocean Virome data identified niche-specialised AMGs that contribute to depth-stratified host adaptations [23]. Given that microbes drive global biogeochemical cycles, and a large fraction of microbes is infected by viruses at any given time [24], viral-encoded AMGs must play important roles in global biogeochemistry and microbial metabolic evolution.

Mangrove forests are the only woody halophytes that live in salt water along the world’s subtropical and tropical coastlines. Mangroves are one of the most productive and ecologically important ecosystems on earth. The rates of primary production of mangroves equal those of tropical humid evergreen forests and coral reefs [25]. As a globally relevant component of the carbon cycle, mangroves sequester approximately 24 million metric tons of carbon each year [25, 26]. Most mangrove carbon is stored in soil and sizable belowground pools of dead roots, aiding in the conservation and recycling of nutrients beneath forests [27]. Although mangroves cover only 0.5% of the earth’s coastal area, they account for 10–15% of the coastal sediment carbon storage and 10–11% of the total input of terrestrial carbon into oceans [28]. The disproportionate contribution of mangroves to carbon sequestration is now perceived as an important means to counterbalance greenhouse gas emissions.

Despite the ecological importance of mangrove ecosystem, our knowledge on mangrove biodiversity is notably limited. Previous reports mainly investigated the biodiversity of mangrove fauna, flora and bacterial communities [29–31]. Particularly, little information is available about viral communities and their roles in mangrove soil ecosystems [32, 33]. In view of the importance of viruses in structuring and regulating host communities and mediating element biogeochemical cycles, exploring viral communities in mangrove ecosystems is essential. Additionally, the intermittent flooding of sea water and resulting sharp transition of mangrove environments may result in substantially different genetic and functional diversity of bacterial and viral communities in mangrove soils compared with those of other systems [34]. Therefore, in this study, we utilised high-depth sequencing and virome-specific bioinformatics tools to explore viral communities and their possible roles in mangrove ecosystems.

## Results

### Overview of mangrove soil viromes

**Fig. 1:**
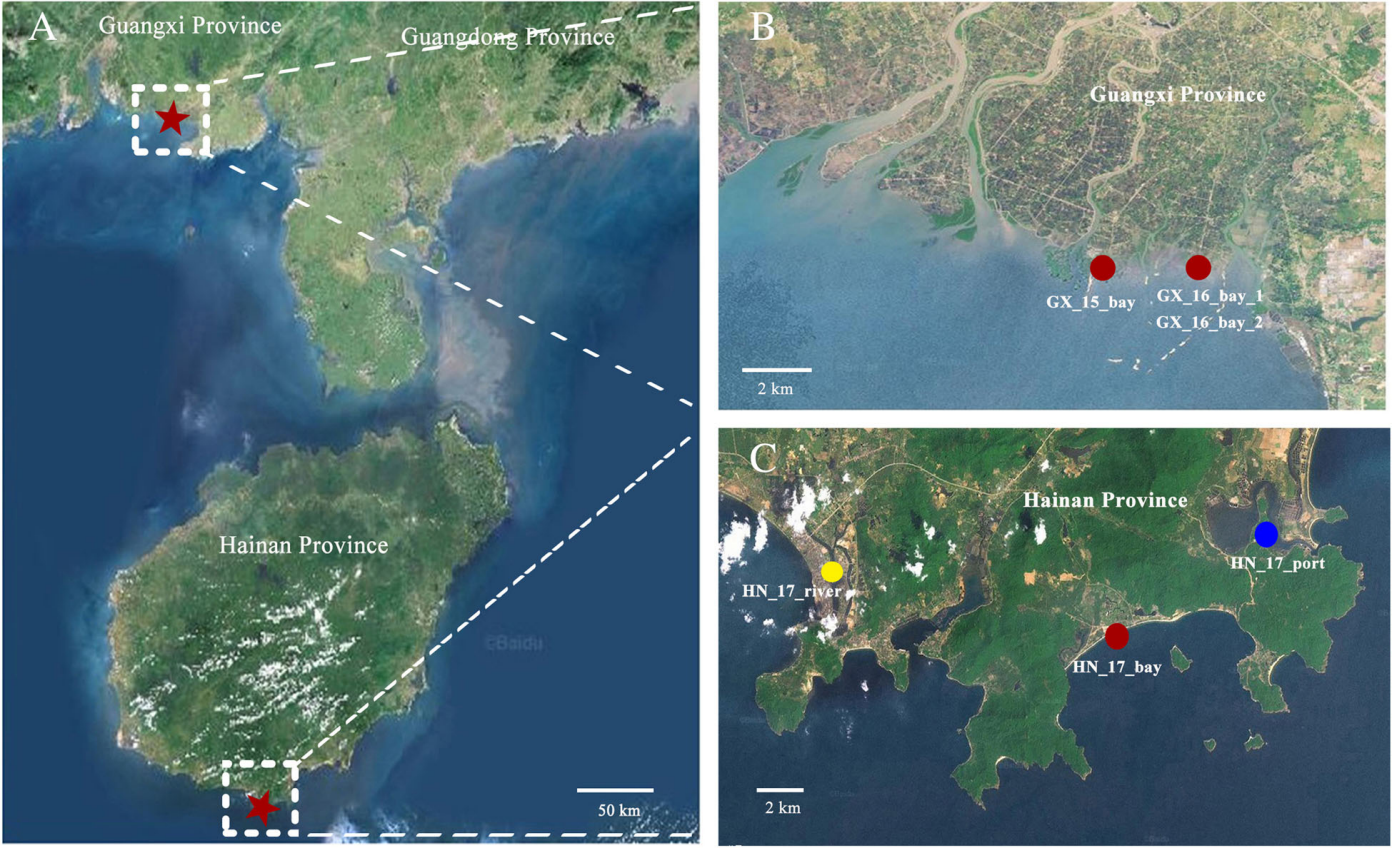
Sampling sites of mangrove soils. **a** Satellite image of sampling sites in Guangxi and Hainan Provinces, China. Mangrove soil sampling sites are indicated with red stars. **b** Satellite image of sampling sites in Guangxi Province. **c** Satellite image of sampling sites in Hainan Province. For panels B and C, sampling sites are marked based on their sampling habitats, i.e., red, yellow and blue dots for bay, river and port, respectively. Refer to the ‘Methods’ section for details

To investigate the mangrove soil viral community structure and to reveal the genetic and functional diversity of mangrove soil viruses, six soil samples were collected from three different mangrove habitats (bay, river and port) in two distant areas (Guangxi and Hainan Provinces, China) for a period of 2 years (2015 October–2017 March) (Fig. 1 and Additional file 1: Table S1). Additional file 1: Table S1 summarises the physical and chemical properties of the soils. The mangrove soil bacterial and viral communities were studied with 16S rRNA gene analysis and viromes, respectively. For virome profiling, virus-like particles were purified for each sample. The encapsidated viral DNA was extracted, randomly amplified and sequenced using an Illumina HiSeq 4000 platform, yielding 2.27–3.99 billion base-pair clean reads available for each virome. The base-pair clean reads were subsequently filtered and assembled into several hundred thousand contigs for each virome (Additional file 1: Table S2). A range of 21,775–133,306 open reading frames (ORFs) were predicted with MetaGene from contigs for each virome (Additional file 1: Table S2). To ensure that no exogenous DNA contaminated the viromes, the purified virus particles were sieved through 0.22 μm filters and treated with DNase I with extended digestion times. The absence of free and contaminating bacterial DNA was validated via *polymerase chain reaction* (PCR) amplification of the bacterial 16S rRNA gene with universal primers 27F/1492R (Additional file 1: Figure S1). In addition to removing cellular DNA during sample preparation, virome reads were also in silico filtered a posteriori to identify and remove any non-viral signal (refer to the ‘Methods’ section for details, Additional file 1: Table S3).

### Morphological and taxonomic diversities of mangrove soil viral communities

Examination of purified virus particles via TEM revealed four major morphological types of mangrove soil viruses, that is, non-tailed spherical viruses and three types of tailed viruses (myoviruses with contractile tails, siphoviruses with long and non-contractile tails and podoviruses with short tails) (Additional file 1: Figure S2).

**Fig. 2:**
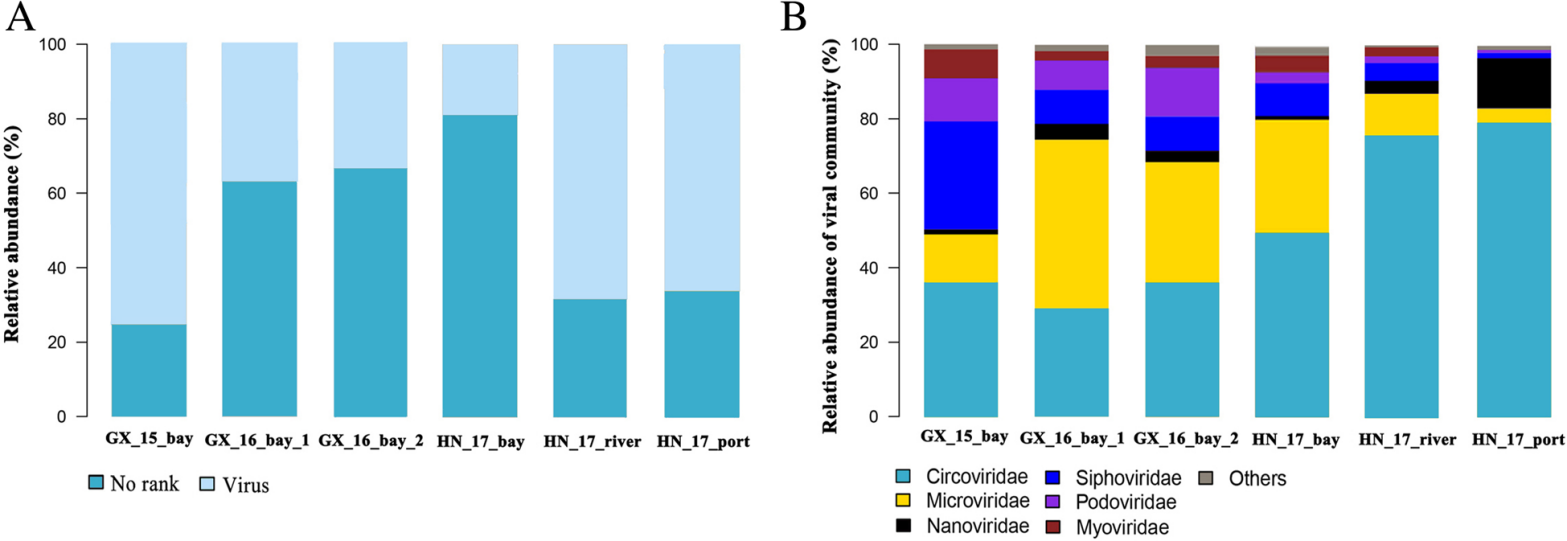
Taxonomic compositions of viral communities in six mangrove soils. **a** Percentage of annotated viral sequences in the virome. **b** Viral taxonomic compositions at the family level. The ‘Others’ category includes 46 different viral families belonging to ssDNA, dsDNA and ssRNA viruses with < 2% relative abundance. MDA was employed to amplify viral nucleic acids, which may over-amplified ssDNA viruses and skewed the viral community’s taxonomic profile

Viral taxonomic affiliations were determined by comparing the predicted virome ORFs against viral sequences from the National Center for Biotechnology Information (NCBI) RefSeqVirus database. The mangrove viruses were largely unknown, as only a proportion of sequences was similar to any sequence in the RefSeqVirus database (Fig. 2a). A total of 51 viral families belonging to ssDNA, dsDNA and ssRNA viruses were identified in mangrove soil viruses. In all the virus-affiliated sequences, matches associated with ssDNA viruses were the most common, with primary assignment to the eukaryal ssDNA family *Circoviridae* (naturally infecting birds and mammals), ssDNA bacteriophage family *Microviridae* and eukaryal ssDNA family *Nanoviridae* (naturally infecting plants) (Fig. 2b). dsDNA viruses, which are most affiliated within the order *Caudovirales* (naturally infecting bacteria and archaea), accounted for the second largest fraction in mangrove soils. Other viral families identified at a relatively high abundance included *Phycodnaviridae*, *Geminiviridae*, *Parvoviridae*, *Inoviridae* and *Mimiviridae*. However, the high presence of ssDNA viruses in the mangrove soil viromes is most possibly due to the bias of multiple displacement amplification (MDA). MDA sufficiently augment the viral DNA for metagenomic analyses but will preferentially amplify genomes of ssDNA viruses and thus skew a quantitative taxonomic profile [35–37]. Therefore, for robust analysis of mangrove soil viruses and reliable ecological interpretations, all the results from these mangrove viromes will be analysed and reported in a qualitative manner hereafter in this study.

A total of 2082 viral species were identified from mangrove soil viromes. Comparative analysis showed that mangrove soils had a similar profile of viral compositions, as 35.5% of viral species were shared by all six viromes and 88% of viral species were shared by at least two viromes (Additional file 1: Figure S3). However, we also identified some environment-specific viral species. For example, five environmental halophages and nineteen mycobacteriophages were unique to bay viromes; they are found in all bay viromes but not in river and port viromes. Several circo-like viruses (e.g., cyclovirus and avian orthoreovirus) and enterobacteriaphage were unique to river virome, whereas no unique viral species was identified in port virome.

### Genetic diversity of mangrove soil viral communities

To assess the diversity and genetic distance among the viruses in the six mangrove soils, phylogenetic analyses were performed on the major viral families of the six viromes by using different marker genes. Virome contigs homologous to each marker (> 65% similarity and > 300 bp aligned nucleic acids) were selected and further clustered (> 95% similarity and 90% coverage) to generate unique viral contigs for phylogenetic analysis of the targeted viral groups.

**Fig. 3:**
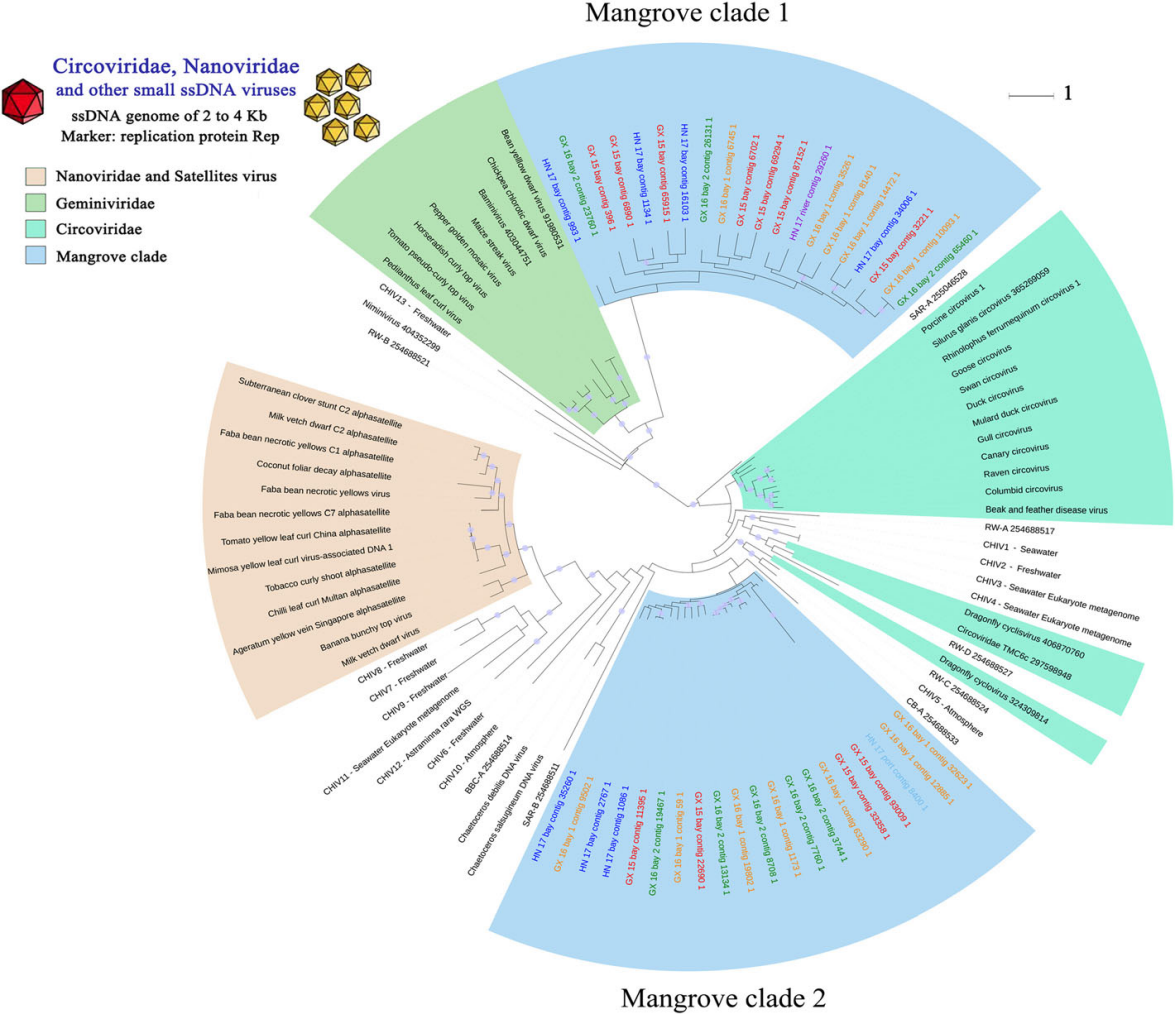
Phylogenetic tree of Circo-like viruses. Viral family-specific protein marker Rep was used to construct phylogenetic trees from mangrove virome contigs and reference sequences for Circo-like viruses. Environmental viral sequences amplified from Reclaimed Water (RW), Chesapeake Bay (CB), Sargasso Sea (SAR) or British Columbia (BBC) were considered as additional references [39, 40], as well as the Rep genes from CHIV [41] and two diatoms viruses (Chaetoceros viruses). Reference sequences are coloured in black, whereas virome contigs are indicated with different colours in the tree. The tree was bootstrapped with 100 subreplicates based on maximum-likelihood methods, and nodes with confidence levels higher than 30% are flagged with circles. The scale bar represents one amino acid substitution per site.

The maximum-likelihood tree was constructed for small ssDNA eukaryotic viruses based on the replication protein Rep, which is conserved in different families of small ssDNA eukaryotic viruses (*Circoviridae*, *Nanoviridae*, satellite viruses, *Chaetoceros* viruses and *Geminiviridae*, designated as Circo-like viruses). A set of 40 unique virome contigs was included in multiple alignments of the reference sequences of Rep protein. As shown in Fig. 3, all virome sequences were distant from known references and environmental metagenomic sequences, forming two separate clades in the tree with no known representative and confirming the undescribed diversity and novelty of this viral family in mangrove soils. The two new clades were distant from each other and included sequences from almost every mangrove samples regardless of the significant differences of environmental conditions and bacterial community structures among these samples. This condition highlights the possible common genetic features of global mangrove viruses.

**Fig. 4:**
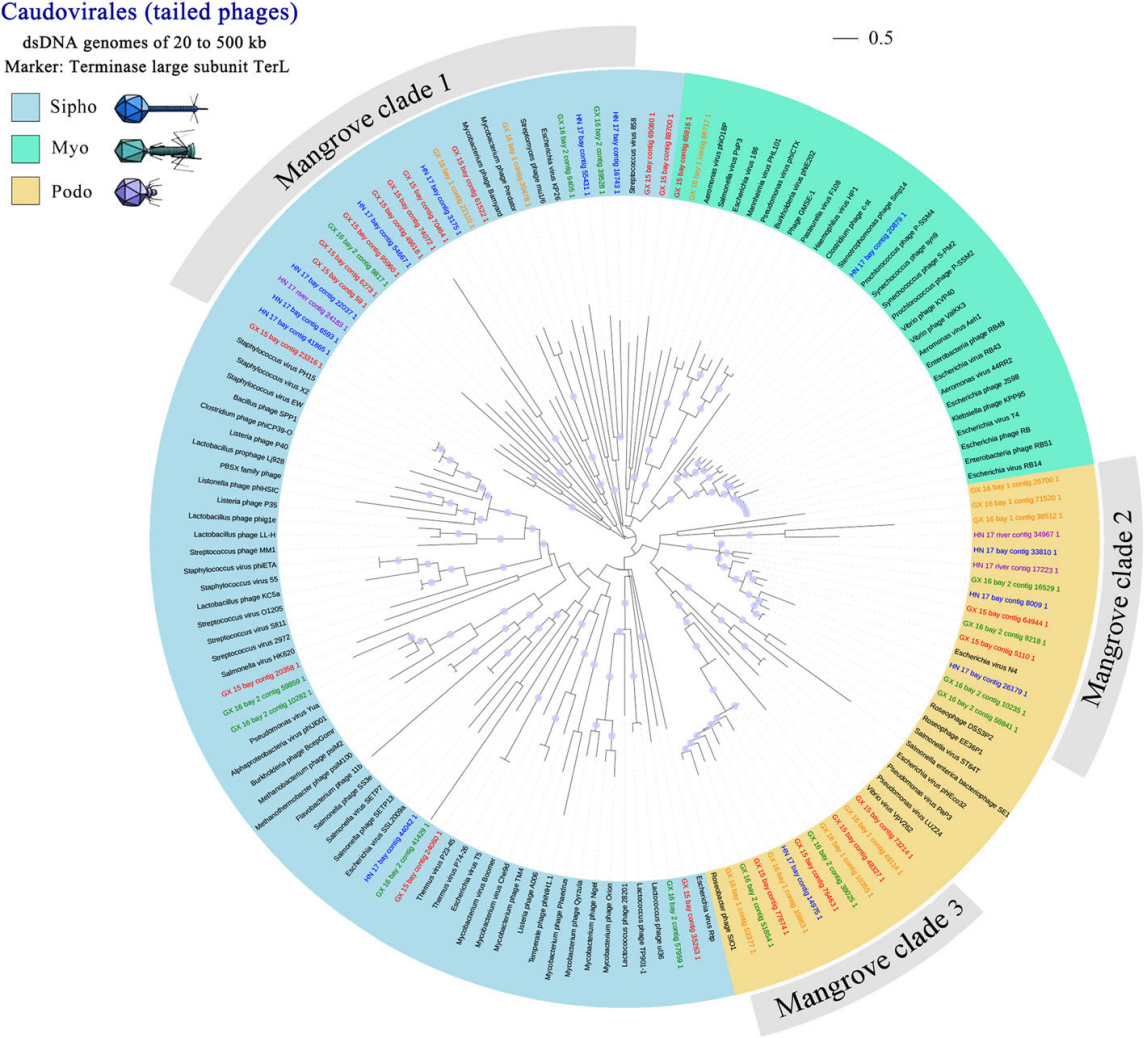
Phylogenetic tree of Caudovirales. Viral family-specific protein markers including TerL were used to construct phylogenetic trees from mangrove virome contigs and reference sequences for Caudovirales. Reference sequences are coloured in black, whereas virome contigs are indicated with different colours in the tree. The tree was bootstrapped with 100 subreplicates based on maximum-likelihood methods, and nodes with confidence levels higher than 30% are flagged with circles. The scale bar represents half amino acid substitution per site

*Caudovirales* form an order of tailed dsDNA phages and are usually the most retrieved dsDNA viruses in the environment. Their diversity can be assessed with a gene coding for terminase large subunit (TerL); 60 unique virome contigs were used in phylogenetic analyses. As shown in Fig. 4, mangrove viral sequences were widely distributed throughout the tree, with most of the sequences affiliated within *Sipho*- and *Podoviridae* families. Although *Caudovirales* are the best-studied virus group to date, most mangrove *Caudovirales* were phylogenetically distant to known reference sequences and formed three mangrove clades within *Sipho*- and *Podoviridae* families. This result highlights an important uncharacterised diversity for Caudovirales in mangrove soils. Six viral reference sequences are included in the three mangrove clades, providing clues to determine the hosts of phages from these clades. For example, mangrove clade 1 included three references of *Actinomyces* phages, indicating that members of this clade may mainly infect *Actinomyces*. Consistent with Rep phylogenetic tree, all the clades included representatives from different mangrove samples. These results further support our hypothesis that the mangrove viral genetic pool maybe consistent across mangrove habitats.

### Abundant auxiliary carbohydrate metabolic genes in mangrove viruses

**Fig. 5:**
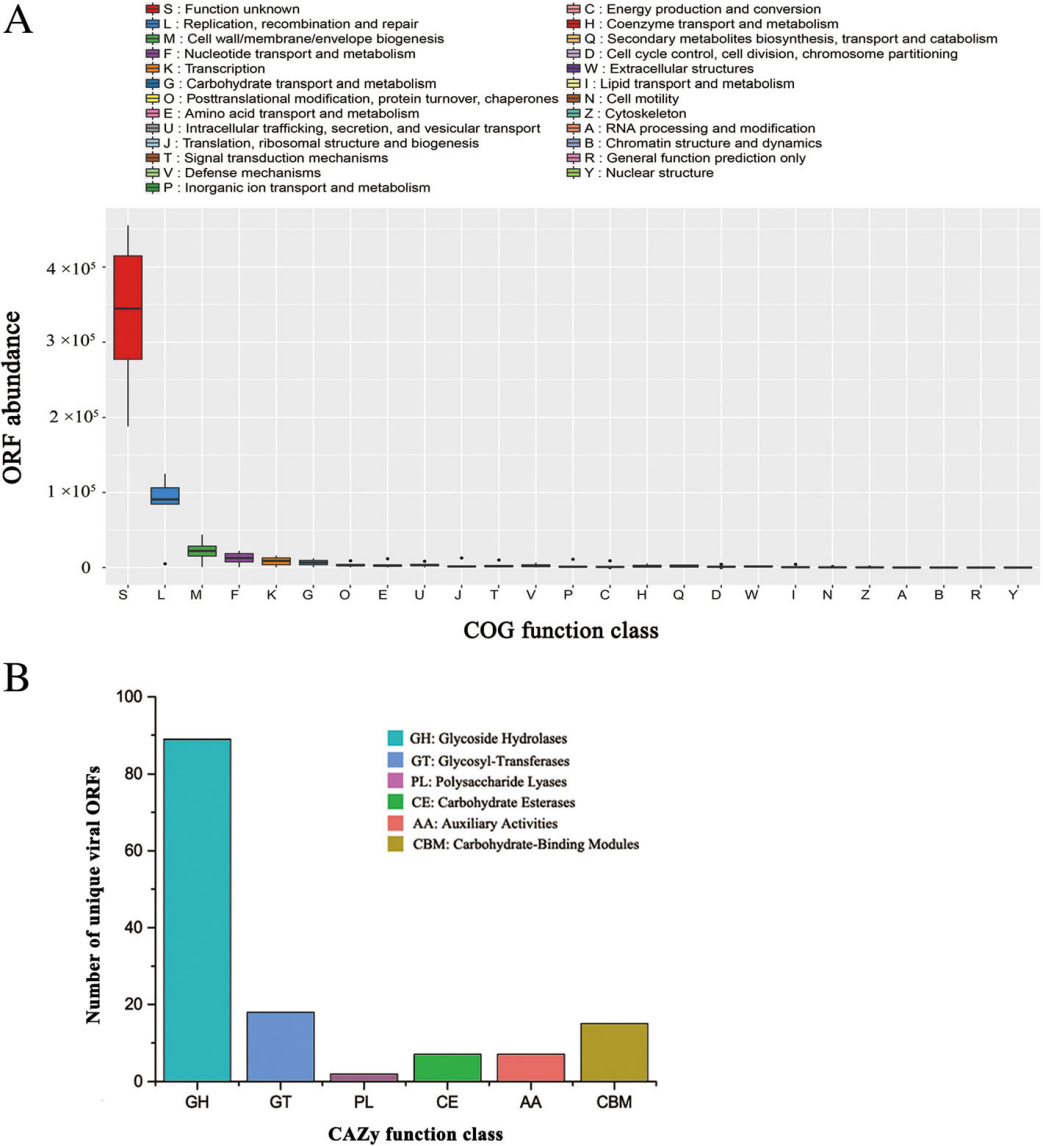
Abundant auxiliary carbohydrate–metabolism genes in mangrove viruses. **a** Functional annotation of mangrove viromes. Functional annotation was performed by Blastp comparisons of predicted ORFs with eggNOG database (*E*-value < 1e^−5^). COG function class was ordered according to their hit frequency. Boxplots are constructed with the upper and lower lines corresponding to the 25th and 75th percentiles, respectively, whereas outliers are displayed as points. **b** Annotation of viral carbohydrate–metabolism-related ORFs in the CAZy database

To investigate the functional diversity of mangrove viruses, clusters of orthologous group (COG) annotation of six viromes was performed by comparing the predicted viral ORFs against the database of evolutionary genealogy of genes: Non-supervised Orthologous Groups (eggNOG database). As expected, most ORFs were poorly annotated, indicating the abundance of largely uncharacterised viral genes in mangrove soils (Fig. 5a). Although the annotated ORFs were widely distributed in all COG function classes, they are notably enriched in a limited number of classes, as proven by the top five assigned function classes accounting for the majority of annotated viral ORFs. The category of functional ORFs showed strong consistency with conventional viral functions, such as ‘replication, recombination and repair’, ‘cell wall/membrane/envelope biogenesis’, ‘nucleotide transport and metabolism’ and ‘transcription’, which are critical for the reproduction and survival of viruses. Remarkably, compared with other auxiliary metabolic functions, ‘carbohydrate transport and metabolism’ was notably over-represented in mangrove viromes (Fig. 5a).

After the removal of redundant ORFs, 705 unique carbohydrate metabolism-related ORFs were identified in mangrove viromes. A total of 138 ORFs were further identified as carbohydrate-active enzymes (CAZymes) by the dbCAN server based on the recognition of the CAZyme signature domain (Fig. 5b). According to the CAZy and NCBI non-redundant protein (NR) database annotation results, the 138 ORFs belong to 27 CAZyme genes, with most of the ORFs affiliated to CAZymes with polysaccharide hydrolase activities (Table 1), implying the possible important roles of these CAZyme genes in the decomposition of mangrove soil organic carbons.

**Table 1:** Annotated auxiliary CAZymes from mangrove soil viruses

Viral auxiliary CAZymes	
GH families	
Chitinase (GH119)	
Xylanase (GH10)	
Alpha-amylase (GH13, GH57)	
Mannanase (GH26)	
Endoglucanase (GH119)	
Cellobiosidase (GH119)	
Beta-glycanase (GH16)	
Alpha-glucanotransferase (GH77)	
Beta-N-acetylhexosaminidase (GH3)	
Glucan 1,3-alpha-glucosidase (GH15)	
Glucan 1,3-beta-glucosidase (GH55)	
1,3-beta-glucanase (GH16, GH17)	
Sugar isomerase (GH58)	
Glucose-6-phosphate isomerase (GH99)	
Trehalase (GH37)	
CE families	
Polysaccharide deacetylase (CE4)	
Pectinesterase (CE8)	
PL families	
Pectate lyase (PL3)	
AA families	
Quinoprotein glucose dehydrogenase (AA12)	
l-sorbosone dehydrogenase (AA12)	
GT families	
Glycosyltransferase (GT2)	
Galactosyltransferase (GT1)	
Rhamnosyltransferase (GT1)	
Glycerate 2-kinase (GT16)	
Alpha,alpha-trehalose-phosphate synthase (GT20)	
Alpha-glucan phosphorylase (GT35)	
Glycogen synthase (GT5)	

**Fig. 6:**
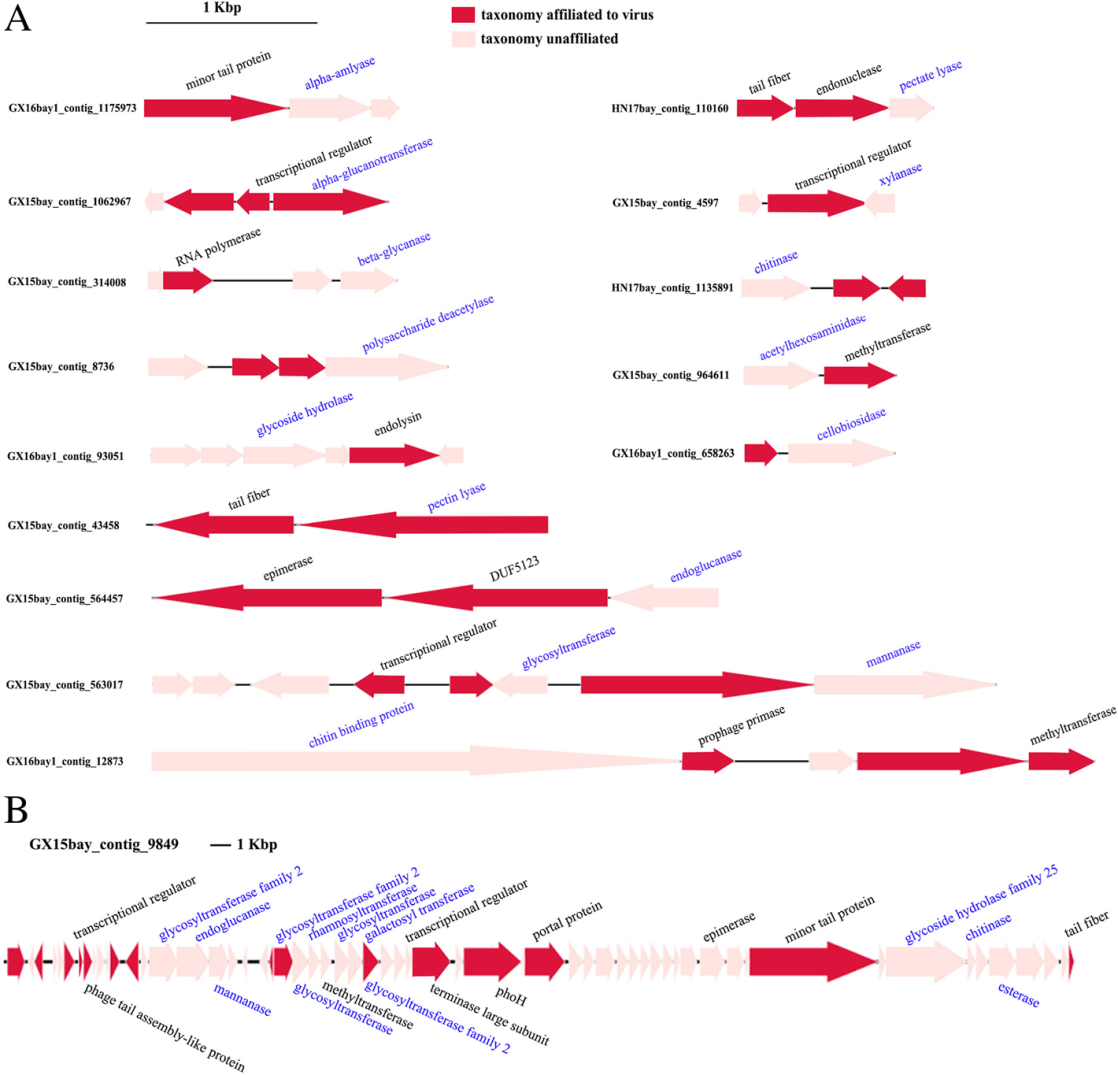
Map analysis of CAZyme-containing contigs. **a** Representative contigs containing CAZyme genes. **b** Map analysis of a complete phage genome containing abundant CAZyme genes. For both plots, CAZyme genes are shown in blue, and other genes are shown in black. Genes are coloured based on superkingdom annotation, i.e., red, viral; light red, unaffiliated

Linkage information or genomic context was used to validate viral AMG identification (Fig. 6). Although we have obtained considerably more clean reads (2.27–3.73 Gbp for each sample, Additional file 1: Table S2) from Illumina Hiseq sequencing than most of the reported viromes, the average length of assembled contigs was relatively small (616–687 bp). We speculated that this result may be largely due to the high abundance and diversity of mangrove soil viruses. Consequently, a large proportion of CAZyme-containing contigs contained a single gene, causing difficulty in characterising the genomic context of CAZymes. Despite these limitations, 16 out of 27 CAZyme genes and 9 out of 15 glycoside hydrolase genes were validated as virus-encoded based on linkage information (Fig. 6). Interestingly, we also have assembled a complete phage genome from mangrove soil viromes (Fig. 6b). This genome approximates in 52 kb length and encodes 59 putative ORFs. Remarkably, auxiliary CAZymes are highly enriched in the phage genome, indicating the high occurrence of CAZymes in mangrove soil viruses.

**Fig. 7:**
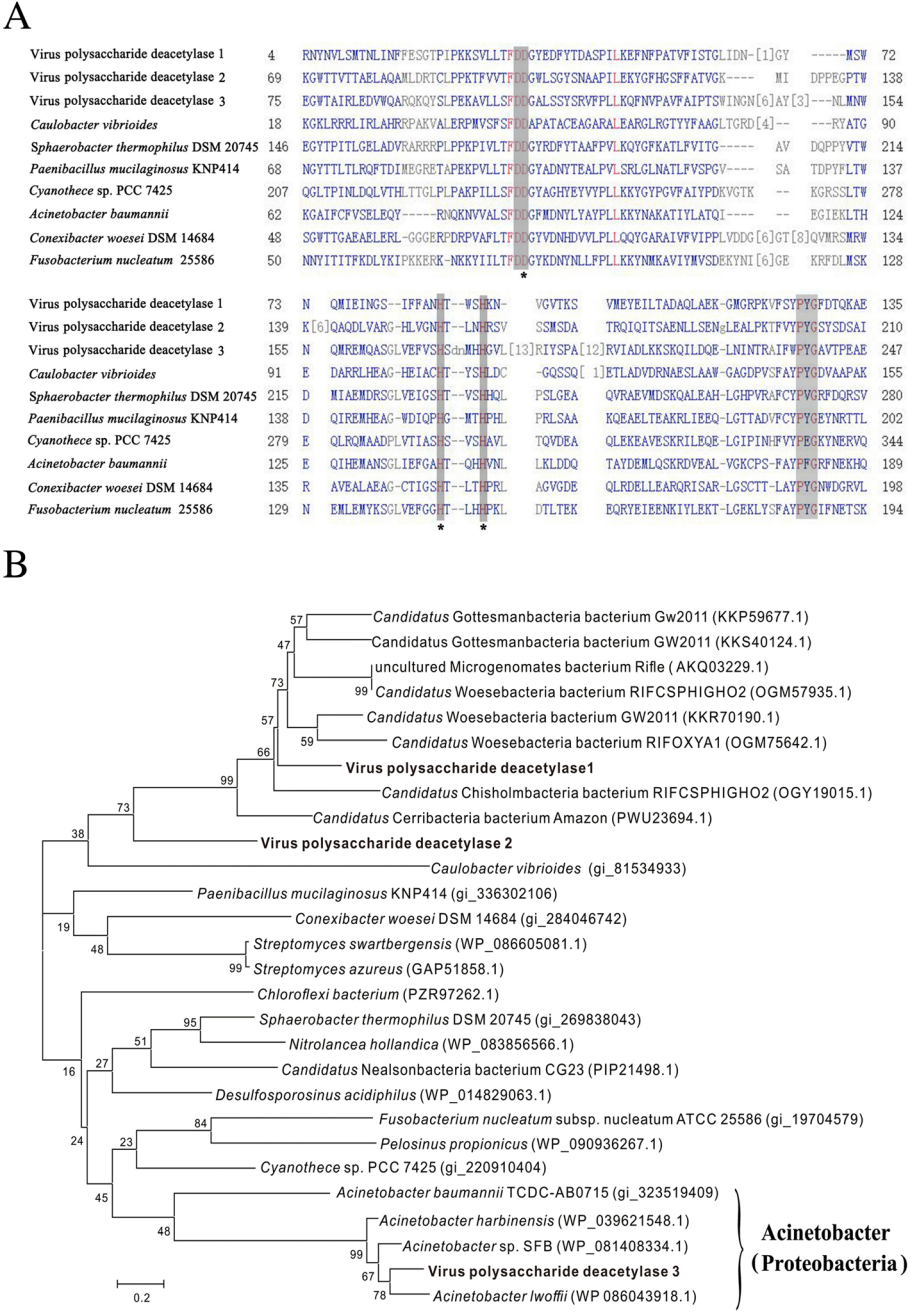
Sequence analysis of viral polysaccharide deacetylases. **a** Conserved motif of viral and bacterial polysaccharide deacetylases. Multiple alignments of viral and bacterial polysaccharide deacetylase proteins revealed the presence of conserved active sites and metal binding sites in all viral sequences, which are essential for their enzymatic activities. Putative active sites are shaded in grey, whereas putative metal binding sites are indicated with asterisk. **b** Phylogenetic analysis of viral and bacterial polysaccharide deacetylases based on amino acid sequence. The tree was constructed using the maximum-likelihood method. Numbers indicating bootstrap values of 500 trials are shown. The scale bar represents 0.2 amino acid substitution per site

Finally, we attempted to identify the hosts of viruses encoding CAZymes based on AMG phylogenetic trees. Putative hosts for 10 CAZymes were predicted (Table 2). For example, phylogenetic analysis showed that viral polysaccharide deacetylase 3 clustered with polysaccharide deacetylases from *Acinetobacter*, indicating that this AMG was possibly derived from phages infecting this bacterium (Fig. 7). However, the hosts of polysaccharide deacetylases 1 and 2 were uncertain, as they clustered with polysaccharide deacetylases from bacteria of different phyla. Notably, viral CAZymes were widespread in phages infecting diverse bacteria of different phyla (Table 2), as exemplified by phylogenetic analysis of alpha-amylase (Additional file 1: Figure S4) and chitinase (Additional file 1: Figure S5). Furthermore, bacterial taxonomic composition analysis showed that the putative hosts for viral CAZymes were among the most abundant bacterial phyla in mangrove soil (Additional file 1: Figure S6), implying the potential enormous impacts of viral CAZymes on mangrove ecosystems.

**Table 2:** Host prediction of CAZymes-containing viruses

Viral auxiliary CAZymes	Predicted hosts (phyla)
Chitinase	Proteobacteria, Actinobacteria, Firmicutes
Alpha-amylase	Actinobacteria, *Candidatus* Saccharibacteria, Proteobacteria
Xylanase	Firmicutes
Mannanase	Actinobacteria
Endoglucanase	Proteobacteria, Actinobacteria
Cellobiosidase	NA^a^
Beta-glycanase	Proteobacteria
Beta-N-acetylhexosaminidase	Proteobacteria
Glucan 1,3-alpha-glucosidase	NA
Glucan 1,3-beta-glucosidase	*Candidatus* Saccharibacteria
1,3-beta-glucanase	Acidobacteria, Planctomycetes
Polysaccharide deacetylase	Proteobacteria
Pectinesterase	NA
Pectate lyase	NA

^a^NA, no host was predicted for this CAZyme

## Discussion

### Identification of newly described viral clades

In contrast to our current understanding of marine viral communities, the soil virome and its function in terrestrial ecosystems have remained relatively understudied [32]. Among approximately 100 distinct viromes reported to date, only eight soil viromes have been described in literature, and none of these viromes was found in mangrove soils, suggesting the severely underestimated and undersampled viral diversity in soil ecosystems, especially in mangrove soil ecosystems [33, 38]. Indeed, our study showed that mangrove soil viruses are largely unidentified, as a large proportion of mangrove soil virome sequences was poorly taxonomically affiliated (Fig. 2a), and most of the predicted ORFs featured no homologues in public databases (Fig. 5a). Such a high proportion of unknown viral sequences possibly resulted from the specificity of viruses, which are scarcely represented in the current databases, from mangrove soil ecosystems [10, 32]. Indeed, to our best knowledge, no genome of mangrove soil-derived viruses has been sequenced to date, highlighting the lack of knowledge and reference sequences for viruses of mangrove environments.

Phylogenetic analyses of the major viral groups (circo-like viruses and *Caudovirales*) highlighted a remarkable diversity and previously unknown viral clades. Notably, we identified two new mangrove clades with clear separation from known references and environmental metagenomic sequences in the Circo-like virus phylogenetic tree constructed from Rep protein (Fig. 3). Thus far, the astounding diversity of Circo-like virus has been uncovered from metagenomic studies [39–42]. However, the exact evolutionary relationships among these viruses remain obscure. A previous study has demonstrated that chimeric ssRNA and ssDNA viruses (CHIVs) blur the evolutionary borders between the major groups of eukaryotic ssDNA viruses by capturing the capsid protein gene from RNA viruses and replacing Rep genes with distant counterparts from diverse ssDNA viruses [41]. Interestingly, the two new mangrove clades were distant from known references and CHIVs and showed distinction from each other. As indicated in the phylogenetic tree, the mangrove clades that were intermediates between *Circoviridae*, *Nanoviridae* and *Geminiviridae* may provide additional clues to reveal the exact evolutionary relationships among these viruses. According to the genetic distance with references and animal and plant species inhabiting in mangroves, viruses of mangrove clade 1 most possibly infect mangrove trees, whereas those of clade 2 infect mangrove animals, such as crabs, shrimps, fishes and birds. Phylogenetic trees drawn from TerL also spotlighted the significant diversity and novelty of mangrove *Caudovirales* (Fig. 4). Although mangrove Caudovirales are widely distributed among the three-tailed phage family, most of the mangrove Terl sequences formed three novel clades with high internal diversity within *Sipho*- and *Podoviridae* families, highlighting an important uncharacterised diversity for Caudovirales in mangrove soils.

### Mangrove soil viruses may share a common genetic pool

Despite the diverse and far-from-reference characteristics of the mangrove virome sequences, the protein sequences of marker genes are relatively similar among the six-mangrove soil viromes. In addition, all the newly identified mangrove clades of Circo-like viruses and Caudovirales contained sequences from nearly every mangrove samples (Figs. 3 and 4, respectively) regardless of the significant differences of environmental factors (Additional file 1: Table S1) and the bacterial community structures (Additional file 1: Figure S6) between these samples. These mangrove viral communities are thus possibly composed of evolutionarily close viral genotypes and differ primarily in terms of community compositions. In contrast to the mixing/connectivity properties of aquatic systems, soil habitats are intrinsically heterogeneous and diverse [32]. The spatial heterogeneity of soil structure and the resulting lack of connectivity of individual ‘island’ microbiomes within soil aggregates promote parallel microbial evolution trajectories. Such parallel evolutionary events are bound to increase local microbial diversities [43, 44]. However, mangrove soils are intermittently flooded with marine water. Consequently, the ‘island’ microbiomes can, to a certain extent, mix and diminish parallel evolutionary effects. Therefore, we speculate that the similar genetic features of mangrove soil viruses are probably due to the mixing and connectivity effects of marine tides; this assumption also supports the former hypothesis that viral diversity can reach high levels on a local scale but is relatively limited globally [45–47].

### Co-influences of marine and fresh water on mangrove soil viral community

Previous studies have shown that soil viromes demonstrate a distinct feature with aquatic viromes [48, 49]. Consistently, we also identified a number of typical soil viruses in mangrove soils. For example, nine phages infecting *Rhizobium* were widely distributed in six mangrove soil viromes. *Rhizobium* are important for soil ecosystems, as they can undergo endosymbiotic nitrogen-fixing association with plant roots in soils [50]. Notably, unlike other soil systems, mangrove soils occupy the interface of terrestrial and marine systems. In particular, mangrove ecosystems possess the unique feature of intermittent flooding with seawater. Mangroves also constantly receive fresh water from river outflow and sanitary wastewaters. Therefore, mangrove soil viromes are possibly multiple-shaped or affected by soil, marine and fresh water systems. Deeper inspection of viral genotypes revealed notable marine signatures. Phages known to infect typical marine bacteria were widely present in the mangrove soil samples. For example, four dsDNA *Pelagibacter* phages were identified at high relative abundances in six mangrove soil viromes (the actual relative abundance of dsDNA viruses maybe higher, as they were not over-amplified by MDA), including pelagiphage HTVC010P, which infected ‘*Candidatus* Pelagibacter ubique’ of the SAR11 clade, the most abundant bacterium in surface seawater around the world [51]. In a more extensive metagenomic investigation including samples from coastal and open ocean areas, pelagiphage HTVC010P was proposed as one of the most abundant virus subfamilies in marine environments, in which 38.8% of successfully assigned reads were assigned to HTVC010P [52]. High relative abundance of *Celeribacter* phage P12053L was found in mangrove soil viromes. *Celeribacter* phage P12053L, a lytic dsDNA phage infecting bacteria of the *Roseobacter* clade, was isolated from seawater and collected off the coast of the Yellow Sea [53], providing further evidence of the impacts of marine water on mangrove soil viromes.

In general, relatively few impacts of freshwater were observed in mangrove soil virome, which agreed with the general environmental characteristics of our sampling sites. High salinity indicated that the sampling site was more similar to typical marine environments than freshwater. Numerous phages infecting enterobacteria and other mammalian pathogens were identified in the mangrove soil viromes. Similar to their pathogen hosts, the occurrence of these non-marine phages in mangrove soil viromes probably results from freshwater transfer from the river or sanitary wastewaters; such process is highly influenced by intense human activities. Collectively, our results showed that mangrove soil viromes are mainly affected by marine waters, with less influence coming from freshwater.

### Mangrove soil viruses may directly manipulate mangrove carbon cycling

Thus far, most viral AMGs are identified from marine environments [22], and limited information is known about viral AMGs in soils [32, 33]. As soil and ocean are disparate ecosystems with unique ecological drivers, soil viral AMGs may be distinct from those identified in marine viruses. However, to our best knowledge, only three viral AMGs (*trz*N, *pho*H and *RNR*) are reported to date in soil ecosystems. *trz*N, a gene encoding chlorohydrolase required in atrazine catabolism, was identified from phages in atrazine-contaminated soils [54]. *trz*N possibly improves viral capacity to produce more progeny under resource limitations (e.g., where atrazine may be an alternative or sole C and N source). The *pho*H gene (phosphate regulation gene) and RNR gene (encoding for ribonucleotide reductase) were found in the virome of Namib Desert hypoliths [55]. The prevalence of *pho*H in the hypolithic system suggested a significant function of *pho*H in phosphate acquisition, whereas the high abundance of *RNR* gene may be advantageous for viruses in nutrient-limited environments.

Viral AMGs for carbon metabolisms have been extensively investigated in marine environments; most of them are involved in central carbon metabolism to facilitate viral replication [22]. The current paradigm from marine studies is that central carbon AMGs shift host microbial metabolism to mimic a state of starvation. In this model, virally encoded glycogen synthase disrupts host glycolysis and directs host metabolism away from amino acid biosynthesis and towards pathways favouring phage replication [22, 56]. Recently, carbon AMGs relevant to carbohydrate metabolism were identified in bovine rumen viromes; these AMGs include five glycosidic hydrolases (beta-glucosidase, alpha-glucosidase, alpha-amylase, maltooligosyltrehalose, trehalohydrolase and endoglucanase) [56]. Although the implication of viral-encoded glycosidic hydrolase in virus–host interaction remains unknown, it is proposed that rumen virus-encoded glycosidic hydrolases potentially augment the breakdown of complex carbohydrates to increase energy production and boost host metabolism during viral infection [56]. Consistently, all the five rumen virus-encoded glycosidic hydrolases (beta-glucosidase, alpha-glucosidase, alpha-amylase, trehalase and endoglucanase) were also identified in mangrove soil viruses in our study. Moreover, we identified more novel auxiliary carbohydrate metabolism genes in mangrove viruses, including glycoside hydrolases, glycosyl transferases, polysaccharide lyases and carbohydrate esterase (Table 1). This study is the first to report such viral AMGs in soil; most of them were never reported in viruses before. Interestingly, most viral carbohydrate metabolic genes belong to CAZymes with glycoside hydrolase activities (Fig. 5b), indicating that mangrove soil viruses primarily participate in the decomposition of organic carbon.

Mangroves are among the most carbon-rich biomes, accounting for 11% of the total input of terrestrial carbon into oceans [25]. In mangrove ecosystems, a large proportion of the organic carbon is stored as large pools in soils, dead plants and animals in the form of complex carbohydrates. Complex carbohydrates or polysaccharides, such as cellulose, xylan, pectin, starch, alginate, mannan and chitin, are major components of plant cell walls, crustacean shells and intercellular spaces and are highly difficult to degrade [57]. Thus, the biolysis of complex polysaccharides in soils and organism debris is essential for mangrove biomass recycling and critical in local and global carbon cycles. The biodegradation of polysaccharides is a complex process that requires the participation of multiple enzymes [57, 58]. Significantly, mangrove soil viruses encode abundant genes of CAZymes, including core hydrolysis enzymes (cellobiosidase, xylanase, chitinase, alpha-amylase, mannanase and endoglucanase) and auxiliary enzymes (polysaccharide deacetylase, pectinesterase and pectate lyase), that are essential for the degradation of various polysaccharides, indicating the full-scale capabilities of mangrove soil viruses in the biolysis of complex polysaccharides (Table 1). Molecular evolutionary studies of viral-encoded photosynthesis AMGs showed that viruses obtain and maintain AMGs from within their known host ranges for their own fitness advantages [13]. Here, phylogenetic analysis showed that viral CAZyme genes are diverse and probably derive from phages infecting distinct hosts of different phyla, suggesting that auxiliary carbohydrate metabolic genes may be widespread in mangrove soil viruses (Table 2).

In a marine environment, several of the most important and well-described viral AMGs are photosystem I and II genes that have been acquired by phages infecting photosynthetic marine cyanobacteria [59, 60]. The expressions of these genes during infection boost the photosynthetic output of infected cells and play important roles in marine nutrient and biogeochemical cycles [14]. In contrast to marine microbes, most soil microbes are heterotrophic and acquire carbon and energies by decomposing complex organics [32, 33]. Therefore, viral carbohydrate AMGs possibly facilitate hosts to decompose and utilise complex carbohydrates and thus boost viral replication in soil ecosystems, similar with the functions of photosynthetic AMGs in marine environments.

### Methodological Considerations and Limitations

The development of metagenomic approach provides a powerful tool for cultivation-independent investigation of viral communities across ecosystems. However, the procedure starting from sample collection, sequencing preparation to bioinformatics analysis of the virome is experimental and informatics-challenging, which may induce biases in exploring viral communities.

Conventional solutions for amplifying sufficient viral DNA for metagenomics analyses include MDA and linker amplification (LA) [37]. However, both these methods seriously distort the proportion of dsDNA and ssDNA viruses recovered [36]. The LA method is based on the ligation of dsDNA linker to sheared DNA [37]. As ligation occurs between dsDNA fragments, ssDNA viruses are inefficiently recovered through this method. MDA is biassed to over-amplify circular ssDNA [35], which render the resulting metagenomes non-quantitative. Recently, a new virome library preparation protocol has been developed. This protocol incorporates an adaptase step prior to linker ligation and amplification, making the process efficient for both ssDNA and dsDNA templates [61]. Fortunately, the major findings of our study will be exempt from the known bias of MDA approach, because the most significant part of our results (i.e., phylogenetic analysis, AMG characterisation and viral-host linkage) rely on the non-quantitative description of viral genes. Besides, given that MDA over-amplifies ssDNA viruses, the actual relative abundance of dsDNA viruses may be even higher than that looked like in the viromes, which rendered the finding that mangrove soil viromes are co-influenced by marine and fresh water even more robust.

A major concern of investigating viral AMGs is to exclude the contamination of cellular DNA in viromes. To this end, we have exerted our best efforts to remove cellular DNA completely during sample preparation. However, even the most thorough laboratory processing can also yield contaminated viromes [62]. Thus, a second in silico filtration is required to identify and remove any non-viral signal. Currently, VirSorter is the most frequently utilised informatics tool to ensure that only viral genomic data are included within the virome [63]. The VirSorter pipeline as a virome decontaminator features a precision higher than 98.99%, but its performance in detecting short viral contigs (< 3 kb) is extremely limited, as only about 13% of bona fide viral contigs are recovered when the contig length measures 1–2 kb [63]. As the average contig length of our assembled virome is relatively short (616–687 bp, Additional file 1: Table S2), VirSorter may not be a good choice to detect viral signals. Instead, an alternative read filtering was processed by only selecting reads and contigs that were identified as viral or unknown [64]. This method can remove as much contamination as possible but may also lose a considerable portion of viral signals, as certain viral reads and contigs are similar with microbial DNA or contain portions of microbial DNA. Genomic linkage analysis was further utilised to ensure that the predicted auxiliary CAZymes are bona fide viral sequences (Fig. 6). Collectively, these utilised experimental and informatics methods will ensure that virtually no known cellular signal was considered in our analyses.

## Conclusions

In summary, we systemically explored the viral communities in mangrove soil for the first time. The results revealed extensive diversity and previously unknown viral clades in mangrove soils. Comparative analysis of viral genotypes revealed that mangrove soil viromes are mainly affected by marine waters, with less influence coming from freshwaters. Remarkably, we identified abundant auxiliary CAZyme genes from mangrove soil viruses. Given the global relevance of mangroves in the carbon cycle and the probable widespread of carbohydrate metabolic genes in mangrove viruses, the role of viral carbohydrate AMGs in global carbon cycle can be highly significant. Collectively, our results showed that mangrove soil viruses may directly manipulate carbon cycling through the biolysis of complex polysaccharides, implying the important and diverse roles of environmental viruses than previously suspected.

## Methods

### Sampling site descriptions and sample collection

Samples were collected from mangrove soils in Guangxi and Hainan Provinces, China (Fig. 1 and Additional file 1: Table S1). Guangxi mangrove sampling sites (GX_15_bay, GX_16_bay_1 and GX_16_bay_2) are located in Beibu Bay near Beihai City. These sites feature typical regular diurnal tide patterns and subtropical oceanic monsoon climates. A contiguous and well-preserved mangrove ecosystem, wherein *Aegiceras corniculatum* and *Kandelia candel* are the dominant plant communities, is found in this area due to minimal human activities. Hainan mangrove sampling sites (HN_17_bay, HN_17_river and HN_17_port) are located in Sanya City, north of South China Sea and exhibit irregular diurnal tide patterns and tropical oceanic monsoon climates. HN_17_river sample was collected from mangrove soil in the riverbank of Sanya River, which flows through the urban district of Sanya City. The dominant plant communities include *Avicennia marina* and *Rhizophora apiculata*. HN_17_bay sample was collected from mangrove soil in Yalong Bay, which is the most well-preserved mangrove in Sanya City, wherein *Ceriops tagal*, *Rhizophora stylosa* and *Lumnitzera racemosa* dominate the ecosystem. HN_17_port sample was collected from the mangrove soil of Tielu Port of Sanya City. The main plant communities comprise *R*. *apiculata*, *A*. *marina* and *L*. *racemosa*.

Mangrove soil samples were collected in different mangrove habitats between 2015 and 2017 (Additional file 1: Table S1). Three soil sample replicates were collected from each site at 5–10 cm depth. To prevent human contamination, the top 1–1.5 cm of soil core was carefully removed from all sides. Then, the soil sample replicates were combined and divided into two fractions. One fraction was frozen in dry ice and stored at -80 °C in the laboratory for about 1–6 months until metagenomic analysis. The other fraction was stored at 4 °C for further physicochemical analyses. Temperature, salinity and pH were directly measured in mangrove soils at a depth of 5–10 cm with sensors. Nutrient concentrations, including total nitrite, ammonia nitrogen, total carbon and TOC, were determined at Qingdao Science Standard Testing platform (Qingdao, China) by using standard methods.

### Virus purification

Viruses were purified from soil samples according to the methods described by Williamson et al. but with specific modifications [65]. Briefly, 30 g of soil per sample was first thawed on ice for 6–12 h. Then, the thawed soil sample was suspended in 100 mL of SM solution (100 mM NaCl, 8 mM MgSO_4_·7H_2_O and 50 mM Tris/HCl; pH 7.5), shaken for 30 min at room temperature and centrifuged at 3000×*g* for 15 min at room temperature to precipitate soil particles. The supernatant was harvested and filtered sequentially through 0.45 and 0.22 μm filters. Then, virus particles in the filtrate were enriched using 100 kDa centrifugal ultrafiltration tubes by centrifugation at 4000×*g* until the final sample volume measured less than 1 mL. Virus samples were then examined for purity and morphology under a transmission electron microscope (TEM, JEOL 100 CXII).

### Viral DNA extraction and virome sequencing

Prior to viral DNA extraction, virus concentrates were treated with DNase I (Sangon Biotech, China) at 37 °C for 2 h to remove external-free DNA fragments. The absence of free and contaminating bacterial DNA was validated via PCR amplification of the bacterial 16S rRNA gene with universal primers 27F/1492R. Encapsidated viral DNA was extracted as described by Thurber et al. [66]. To obtain adequate quantity of the viral DNA needed for high-throughput sequencing, MDA was employed to amplify the total viral DNA by using illustra Ready-To-Go GenomiPhi V3 DNA Amplification kit (GE, USA) in triplicates; the resulting products were pooled for further sequencing. For Illumina sequencing, viral DNA was firstly fragmented to approximately 300 bp by an Ultrasonic Cell Disruptor (M220, Covaris) and used as a template to create a metagenome library using the TruSeq DNA Sample Prep Kit (Illumina, San Diego, CA, USA). The prepared DNA library was then sequenced using an Illumina HiSeq 4000 platform at Shanghai Majorbio Bio-pharm Biotechnology Co., Ltd. (Shanghai, China) to generate 150 bp paired-end reads. The viromes are available on the NCBI Sequence Read Archive database with accession numbers SRX3777329, SRX3777330, SRX3777331, SRX3777332, SRX3777333 and SRX3777334 for HN_17_bay, HN_17_port, GX_15_bay, GX_16_bay_1, GX_16_bay_2 and HN_17_river samples, respectively.

### Virome analysis

After high-throughput sequencing, read ends were firstly trimmed to improve read quality with Seqprep (https://github.com/jstjohn/SeqPrep). Then, low-quality reads were removed with Sickle (https://github.com/najoshi/sickle) to obtain clean reads. Read filtering was performed to remove reads associated with contamination by cellular genomic fragments. Clean reads were firstly compared with Blast program against NCBI NR database, IMG/VR and RefseqVirus database (thresholds of 10^−3^ on *E*-value and 50 on bit score) to identify viral, bacterial, archaeal or eukaryotic sequences (Additional file 1: Table S3). The reads, annotated as ‘viral sequences’ or ‘unknown’ (without a significant similarity against the database), were selected as filtered reads set for further analysis. Each sample was then independently assembled using SOAPdenovo from filtered clean reads [67]. Contigs were further filtered by removing non-viral signals (thresholds of 10^−3^ on E-value and 50 on bit score)[64]; ORFs were predicted with MetaGene from the filtered contigs [68]. For viral taxonomic affiliation, each predicted gene was compared with Blastp against the NCBI NR database and RefseqVirus database with a threshold of 10^−3^ on *E*-value and 50 on bit score.

### Phylogenetic analysis of mangrove viruses

Phylogenetic trees of mangrove viruses were generated based on the viral group specific makers, i.e., replication protein Rep for Circo-like viruses and TerL for Caudovirales [42, 69]. The assembled virome contigs were firstly blasted against the reference sequences of markers with blastX, and only contigs with >65% similarity and > 300 bp aligned nucleic acids were selected for phylogenetic analysis. The representative sequences were further clustered with the thresholds of 95% similarity and 90% coverage with CD-Hit software owing to the large number of aligned virome sequences. All target contigs were translated into amino acid sequences and were aligned using MUSCLE software. Then, the gaps and ambiguously aligned positions were deleted. After alignment, the phylogenetic tree was constructed based on maximum-likelihood method by using MEGA with 100 bootstrap replicates. The trees used in the figures were manually edited using iTOL version 4 [70].

### Identification of auxiliary carbohydrate metabolic genes

COG of protein functional annotation of viromes was determined by Blastp comparisons of predicted ORFs with eggNOG database (http://eggnog.embl.de/) with a threshold of 10^−5^ [71]. ORFs affiliated to COG function class of carbohydrate transport and metabolism were further clustered to generate unique carbohydrate metabolic ORFs with CD-Hit. Subsequently, CAZymes from these viral ORFs were identified on the dbCAN web server based on CAZyme family-specific HMMs [72]. ORFs related to carbohydrate metabolism and CAZymes were compared with NCBI NR and Pfam database to determine the best annotation and similarity for each ORF.

For contig map analysis, contigs containing CAZymes were retrieved, and ORFs were identified with MetaGene. The ORFs were then compared with NCBI and Pfam databases for functional and taxonomic annotation based on protein sequences.

### Identification of putative hosts of AMG-containing viruses

Putative hosts of AMG-containing viruses were predicted based on AMG phylogenetic trees. Molecular evolutionary studies of virus-encoded AMGs showed that viruses obtain and maintain AMGs from within their known host ranges [13]. Therefore, AMG phylogeny serves as a powerful approach to predict putative hosts [10, 73]. Viral AMG sequences were compared with the NCBI NR database (blastp, threshold of 50 for bit score and 10^−3^ for *E*-value) to retrieve relevant reference sequences. Protein sequences were aligned with the ClustalW program, and the gaps and ambiguously aligned positions were deleted. the MEGA program was used to generate the phylogenetic tree according to maximum-likelihood method.

### Multiple alignment of polysaccharide deacetylases

Multiple alignment of viral and bacterial polysaccharide deacetylase protein was performed using COBALT. The conserved motifs were identified by searching against NCBI Conserved Domain Database.

## Additional files

Additional file 1: **Figure S1.** Examination of exogenous DNA contamination in the extracted viral DNA. The absence of free and contaminating bacterial DNA was validated by PCR amplification of 16S rRNA gene from viral DNA with universal primers 27F/1492R. Lane M, protein marker; Lane 1- Lane 6, viral DNA of six mangrove soils; Lane 7, positive control. **Figure S2.** Four viral morphotypes observed in the Mangrove soils. (A) siphovirus with long and non-contractile tail, (B) myovirus with contractile tail, (C) podovirus with short tail (D) non-tailed virus. Scale bar: 50 nm. **Figure S3.** Comparison of viral species among six mangrove soil viromes. Venn diagram shows the number of shared or unique viral species of six mangrove soil viromes. **Figure S4.** Phylogenetic analysis of viral and bacterial alpha-amylase based on amino acid sequence. The tree was constructed by the maximum-likelihood method. Numbers indicating bootstrap values of 500 trials are shown. The scale bar represents 0.5 amino acid substitution per site. **Figure S5.** Phylogenetic analysis of viral and bacterial chitinase based on amino acid sequence. The tree was constructed by the maximum-likelihood method. Numbers indicating bootstrap values of 500 trials are shown. The scale bar represents 0.1 amino acid substitution per site. **Figure S6.** Bacterial taxonomic compositions of mangrove samples on Phylum level. **Table S1.** Descriptions of mangrove soil samples. **Table S2.** General features of six mangrove soil viromes. Table S3 Taxonomic affiliation of virome reads. (DOCX 8153 kb)

## Abbreviations

CAZyme: Carbohydrate-active enzyme
AMG: Auxiliary metabolic gene
LA: Linker amplification
TOC: Total organic carbon
PCR: Polymerase chain reaction
MDA: Multiple displacement amplification
NCBI: National Center for Biotechnology Information
ORF: Open reading frame
NCBI NR database: NCBI non-redundant protein database
TerL: Terminase large subunit
COG: Clusters of orthologous group
eggNOG: Evolutionary genealogy of genes: Non-supervised Orthologous Groups
