# Supplementary material for: Diversities and potential biogeochemical impacts of mangrove soil viruses
Source: Microbiome. 2019 Apr 11;7:58. doi: 10.1186/s40168-019-0675-9 (PMC6460857; doi:10.1186/s40168-019-0675-9)
Supplement: Supplementary file 1 — Figure S1. Examination of exogenous DNA contamination in the extracted viral DNA. The absence of free and contaminating bacterial DNA was validated by PCR amplification of 16S rRNA gene from viral DNA with universal primers 27F/1492R. Lane M, protein marker; Lane 1- Lane 6, viral DNA of six mangrove soils; Lane 7, positive control. Figure S2. Four viral morphotypes observed in the Mangrove soils. (A) siphovirus with long and non-contractile tail, (B) myovirus with contractile tail, (C) podovirus with short tail (D) non-tailed virus. Scale bar: 50 nm. Figure S3. Comparison of viral species among six mangrove soil viromes. Venn diagram shows the number of shared or unique viral species of six mangrove soil viromes. Figure S4. Phylogenetic analysis of viral and bacterial alpha-amylase based on amino acid sequence. The tree was constructed by the maximum-likelihood method. Numbers indicating bootstrap values of 500 trials are shown. The scale bar represents 0.5 amino acid substitution per site. Figure S5. Phylogenetic analysis of viral and bacterial chitinase based on amino acid sequence. The tree was constructed by the maximum-likelihood method. Numbers indicating bootstrap values of 500 trials are shown. The scale bar represents 0.1 amino acid substitution per site. Figure S6. Bacterial taxonomic compositions of mangrove samples on Phylum level. Table S1. Descriptions of mangrove soil samples. Table S2. General features of six mangrove soil viromes. Table S3 Taxonomic affiliation of virome reads. (DOCX 8153 kb) [file 40168_2019_675_MOESM1_ESM.docx]

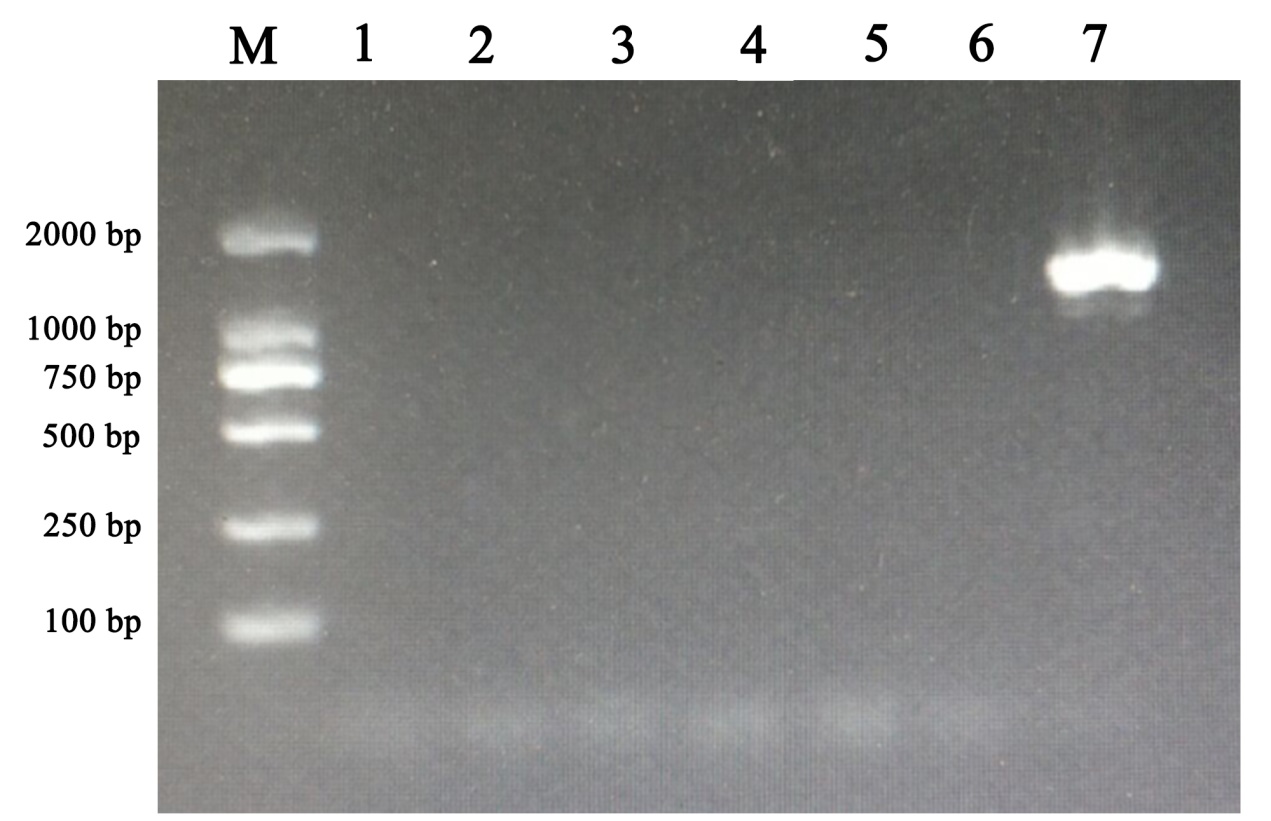


**Fig. S1 Examination of exogenous DNA contamination in the extracted viral DNA.** The absence of free and contaminating bacterial DNA was validated by PCR amplification of 16S rDNA gene from viral DNA with universal primers 27F/1492R.

Lane M, protein marker; Lane 1- Lane 6, viral DNA of six mangrove soils; Lane 7, positive control.


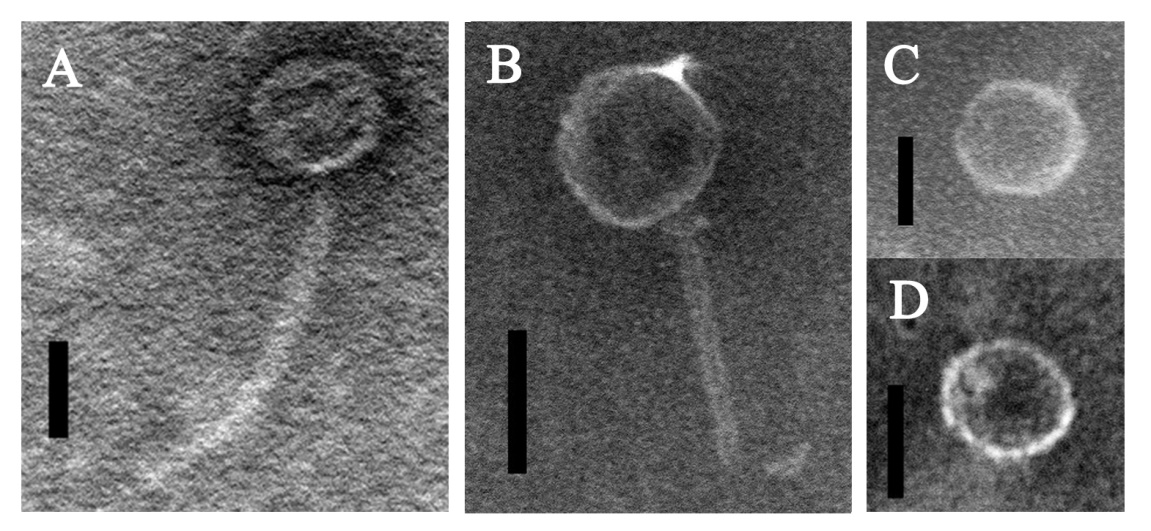


**Fig. S2 Four viral morphotypes observed in the Mangrove soils**. (**A**) siphovirus with long and non-contractile tail, (**B**) myovirus with contractile tail, (**C**) podovirus with short tail (D) non-tailed virus. Scale bar: 50 nm.


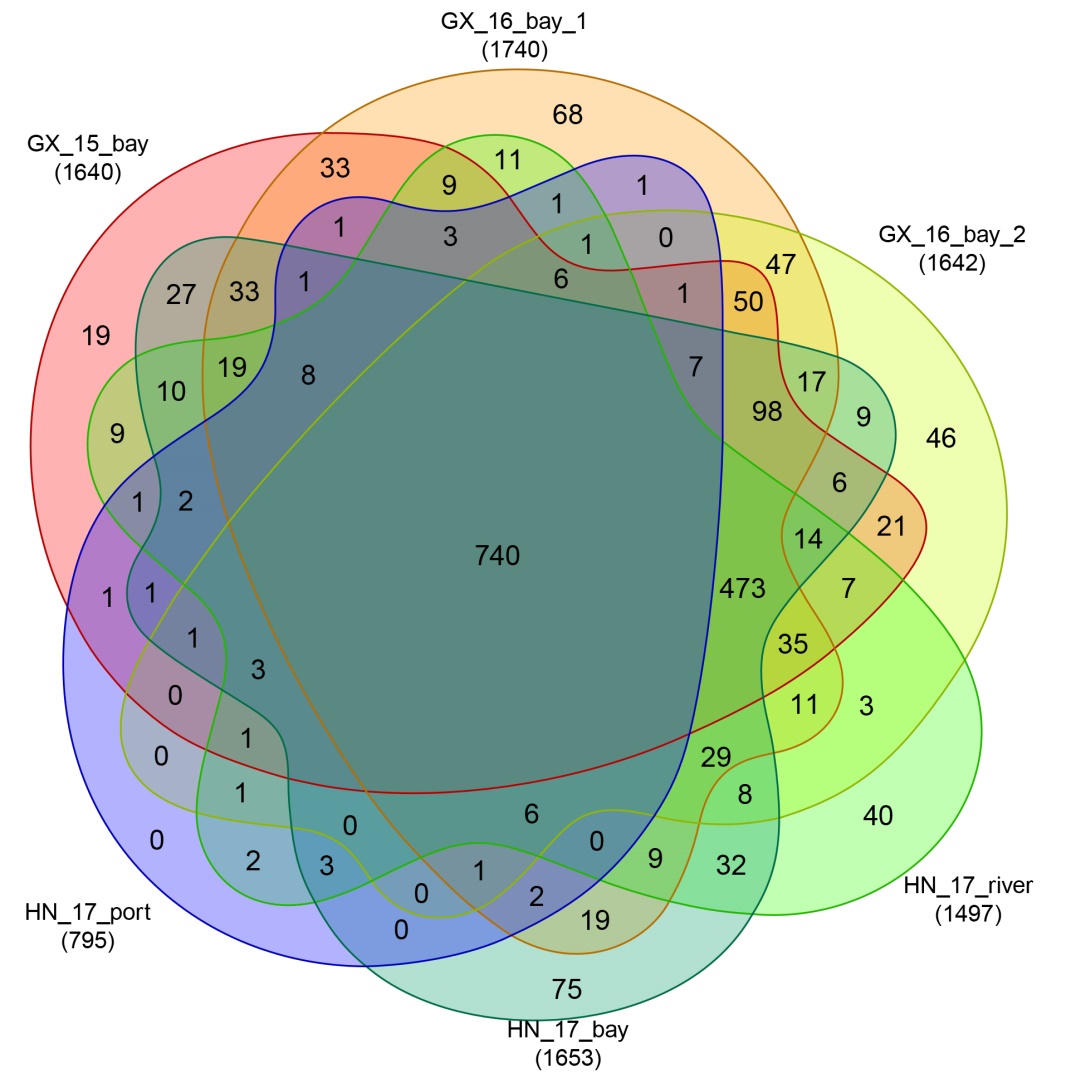


**Fig. S3 Comparison of viral species among six mangrove soil viromes.** Venn diagram shows the number of shared or unique viral species of six mangrove soil vriomes.


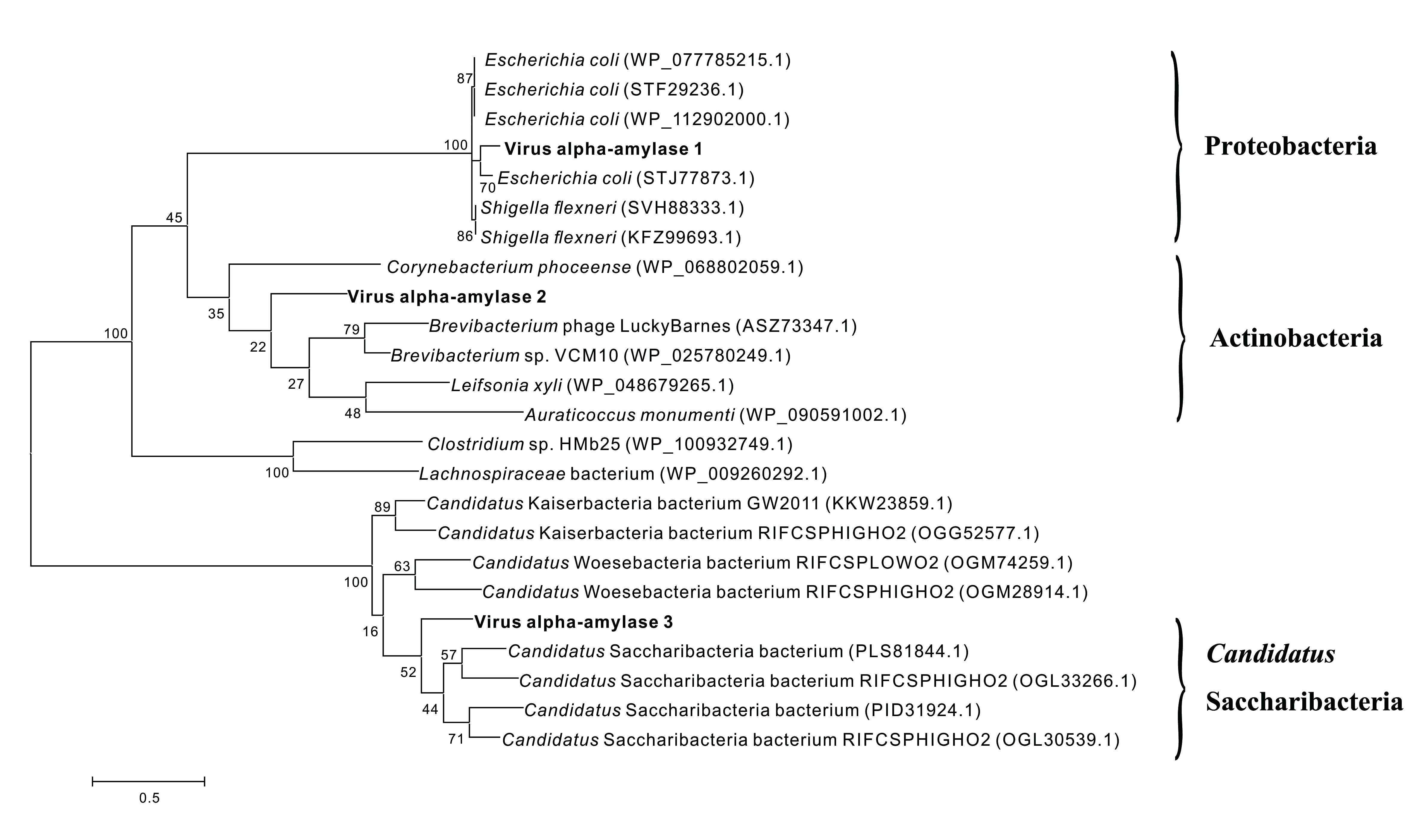


**Fig. S4 Phylogenetic analysis of viral and bacterial alpha-amylase based on amino acid sequence.** The tree was constructed by the maximum-likelihood method. Numbers indicating bootstrap values of 500 trials are shown. The scale bar represents 0.5 amino acid substitution per site.


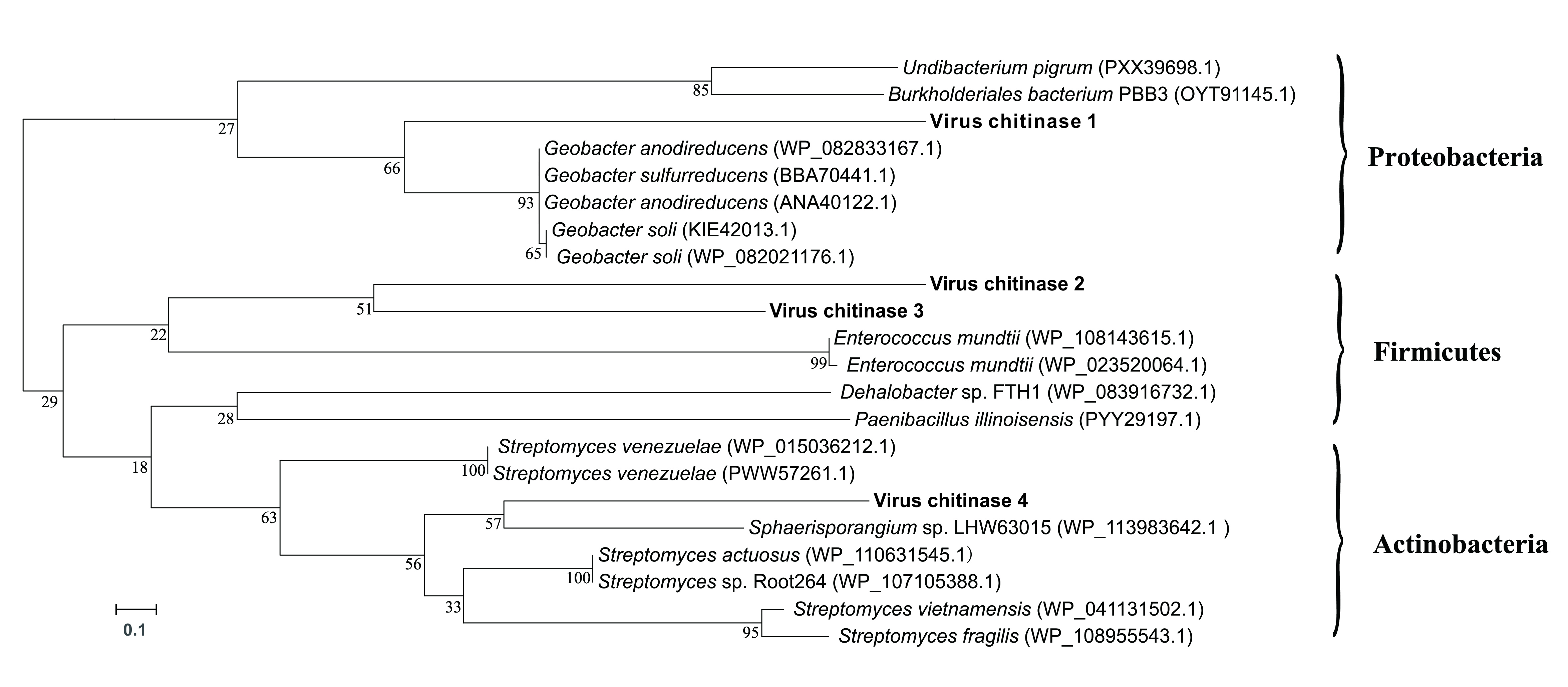


**Fig. S5 Phylogenetic analysis of viral and bacterial chitinase based on amino acid sequence.** The tree was constructed by the maximum-likelihood method. Numbers indicating bootstrap values of 500 trials are shown. The scale bar represents 0.1 amino acid substitution per site.


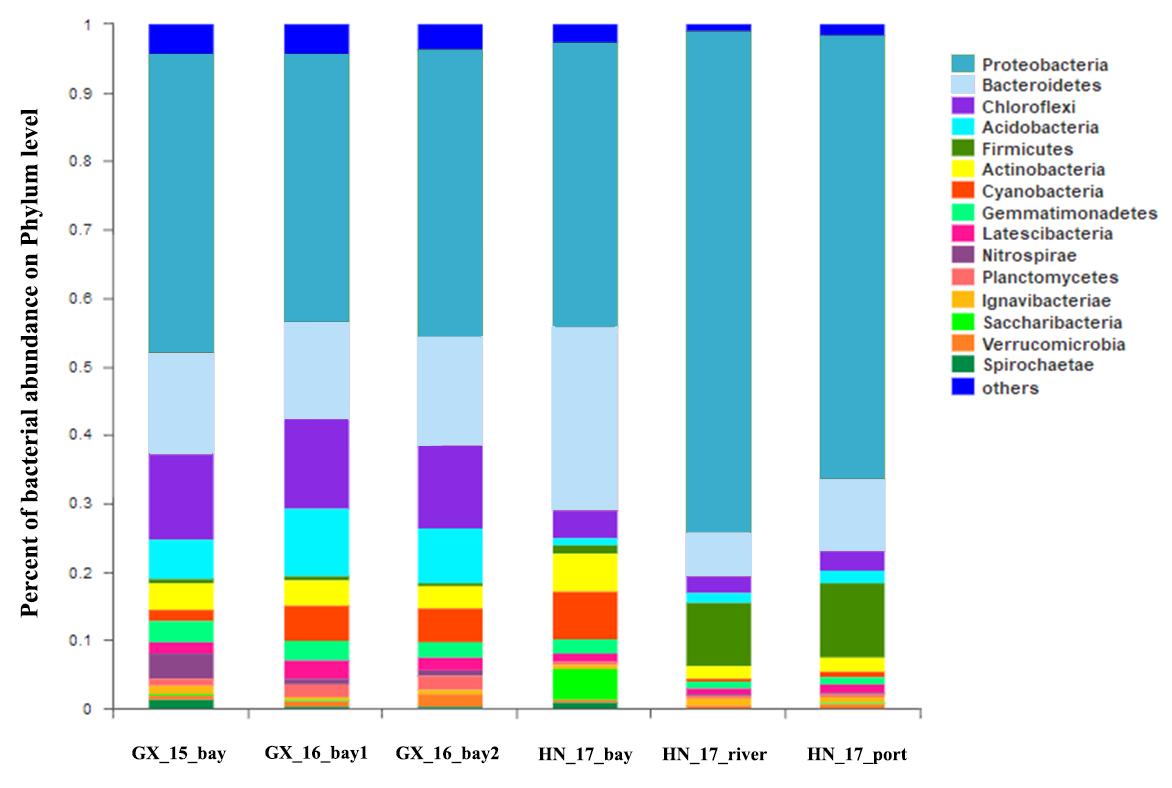


**Fig. S6 Bacterial** **taxonomic compositions of mangrove samples on Phylum level.**

**Table S1 Descriptions of mangrove soil samples**

| Sample | Date | Site | Tide pattern | Dominant flora | Temp(℃) | pH | Salinity(‰) | TN(g/kg) | AN(mg/kg) | TC(g/kg) | TOC(g/kg) |
| --- | --- | --- | --- | --- | --- | --- | --- | --- | --- | --- | --- |
| GX_15_bay | Oct. 2015 | Guangxi, Beibu Bay | regular diurnal tide | *A. corniculatum*  *K. candel* | 24.5 | 7.42 | 18 | 1.62 | 56.1 | 10.08 | 9.83 |
| GX_16_bay_1 | April. 2016 | Guangxi, Beibu Bay | regular diurnal tide | *A. corniculatum*  *K. candel* | 24.1 | 7.85 | 20.1 | 1.48 | 53.2 | 9.62 | 9.15 |
| GX_16_bay_2 | Oct. 2016 | Guangxi, Beibu Bay | regular diurnal tide | *A. corniculatum*  *K. candel* | 24.3 | 7.68 | 20.03 | 1.42 | 49.8 | 8.9 | 8.57 |
| HN_17_bay | Mar. 2017 | Hainan, Yalong Bay | irregular diurnal tide | *C. tagal*  *R. stylosa*  *L. racemosa* | 24.4 | 7.86 | 15 | 1.58 | 63.6 | 8.8 | 8.58 |
| HN_17_river | Mar. 2017 | Hainan, Sanya River | irregular diurnal tide | *A.marina*  *R. apiculata* | 25.5 | 7.66 | 25 | 1.28 | 37.5 | 4 | 1.95 |
| HN_17_port | Mar. 2017 | Hainan, Tielu Port | irregular diurnal tide | *R. apiculata*  *A. marina*  *L. racemosa* | 25.3 | 8.44 | 30.7 | 1.01 | 48.3 | 5.4 | 5.24 |

**Table S2** **General features of six mangrove soil viromes**

| Sample | Sequencing method | Number of clean reads | Total length of clean reads  (Gbp) | Number of contigs | Average length of contigs (bp) | Max. length of contigs (bp) | N50 of contigs (bp) | Predicted ORF number |  |
| --- | --- | --- | --- | --- | --- | --- | --- | --- | --- |
| GX_15_bay | Illumina Hiseq 4000 PE150 | 18317847 | 2.69 | 96266 | 622 | 52012 | 682 | 133306 |  |
| GX_16_bay_1 | Illumina Hiseq 4000 PE150 | 23017146 | 3.37 | 86831 | 687 | 41660 | 796 | 128375 |  |
| GX_16_bay_2 | Illumina Hiseq 4000 PE150 | 15479301 | 2.27 | 67244 | 677 | 33079 | 778 | 97781 |  |
| HN_17_bay | Illumina Hiseq 4000 PE150 | 27144136 | 3.99 | 60419 | 632 | 22379 | 704 | 83154 |  |
| HN_17_river | Illumina Hiseq 4000 PE150 | 18269291 | 2.68 | 46147 | 616 | 27660 | 681 | 61306 |  |
| HN_17_port | Illumina Hiseq 4000 PE150 | 25517949 | 3.73 | 16280 | 683 | 11077 | 837 | 21775 |  |

**Table S3 Taxonomic affiliation of virome reads**

| Taxonomy | GX_15_bay | GX_16_bay_1 | GX_16_bay_2 | HN_17_bay | HN_17_river | | HN_17_port |
| --- | --- | --- | --- | --- | --- | --- | --- |
| No rank | 22.9% | 60.4% | 64.4% | 77.9% | 28.7% | 31.2% | |
| Viruses | 68.2% | 35.4% | 31.0% | 18.2% | 63.6% | 63.3% | |
| Bacteria | 6.1% | 3.6% | 3.3% | 2.8% | 4.9% | 3.5% | |
| Archaea | 1.3% | 0.5% | 0.9% | 0.7% | 1.1% | 0.8% | |
| Eukaryota | 1.5% | 0.1% | 0.4% | 0.4% | 1.7% | 1.2% | |
